# *In Vitro* Anti-AChE, Anti-BuChE, and Antioxidant Activity of 12 Extracts of* Eleutherococcus* Species

**DOI:** 10.1155/2016/4135135

**Published:** 2016-10-10

**Authors:** Daniel Załuski, Rafał Kuźniewski

**Affiliations:** ^1^Department of Pharmacognosy, Collegium Medicum, Jagiellonian University, 9 Medyczna Street, 30-688 Cracow, Poland; ^2^Department of Pharmacognosy, Ludwik Rydygier Collegium Medicum, Nicolaus Copernicus University, 9 Marie Curie-Skłodowska Street, 85-094 Bydgoszcz, Poland

## Abstract

Neurodegenerative diseases are one of the most occurring diseases in developed and developing countries. The aim of this work focused on the screening of the natural inhibitors of AChE and BuChE and antioxidants in* Eleutherococcus* species. We found that the ethanol extracts of* E. setchuenensis* and* E. sessiliflorus* showed the strongest inhibition towards AChE (IC_50_: 0.3 and 0.3 mg/mL, resp.). Among chloroform extracts, the most active appeared to be* E. gracilistylus* (IC_50_: 0.37 mg/mL). In turn, the ethanol extract of* E. henryi* inhibited the strongest BuChE with IC_50_ value of 0.13 mg/mL. Among chloroform extracts,* E. gracilistylus*,* E. setchuenensis*, and* E. sessiliflorus* appeared to be the strongest with IC_50_ values of 0.12, 0.18, and 0.19 mg/mL. HPTLC screening confirmed the presence of inhibitors in extracts. All extracts exhibited anti-DPPH^⁎^ activity and single antioxidants have been identified. To the best of our knowledge, no information was available on this activity of compounds in* Eleutherococcus*. These studies provide a biochemical basis for the regulation of AChE and BuChE and encourage us to continue isolation of active compounds.

## 1. Introduction

Acetylcholinesterase (AChE) and butyrylcholinesterase (BuChE) take part in the termination of nerve impulse transmission at the cholinergic synapses by rapid hydrolysis of acetylcholine (ACh). Excessive hydrolysis of acetylcholine (ACh) and butyrylcholine (BCh) is one of the causes of neurodegenerative diseases, such as Alzheimer's disease (AD), ataxia, or Parkinson's disease.

Alzheimer's disease (AD) is the most common cause of senile dementia in later life. This disease affects from 4% to 10% of the world's population over the age of 65 years. According to the statistics, in Poland, over 250 000 people suffer from AD, while in the United States the number of AD sufferers reached 4 million, and this number increases every single year. Inhibition of these enzymes serves as a strategy for the treatment of Alzheimer's disease (AD), senile dementia, ataxia, myasthenia gravis, and Parkinson's disease [1–4].

Many secondary plant metabolites and several synthetic substances exhibit anti-AChE and anti-BuChE activity; nevertheless, their use is still restricted. Currently used licensed drugs (tacrine, donepezil, and rivastigmine) show selectivity towards one of the two enzymes, which leads to a compensation mechanism and increased activity of the other one. It has been found that these compounds have adverse effects including gastrointestinal disturbances and problems associated with bioavailability. Besides, tacrine is hepatotoxic, and hepatoprotective drugs have to be used simultaneously. Rivastigmine (Exelon) was licensed for use in Europe in 2000. It is well known that synthetic inhibitors just slow the progress of the disease but do not cure it [5, 6].

One of the most intensively studied areas of research has been the investigation of plants with an anticholinesterase action. The increasing incidence of AD has given fresh impetus to the search for novel anti-AChE and anti-BuChE agents. New lead compounds are also needed in the field of pharmacotherapy, phytotherapy, and natural products.

A new source of AChE and BuChE inhibitors can be secondary metabolites present in* Eleutherococcus* species, which are native to Eastern Asia, the Himalayas, and Northeastern Russia. The main secondary metabolites are polyphenols that include eleutherosides B, B1, E, and E1 and are thought to be the pharmacologically most active constituents [7–12]. These species have been used in old Chinese pharmacy for improving human's physical and mental efficiency. People used to believe that they improve cognitive functions, such as learning or memory; thus, scientists now take into account the use of* Eleutherococcus* in neurodegenerative ailments.

Only few papers describing a weak inhibitory effect of* E. senticosus* on the regeneration of neuritis damaged by amyloid*β*(25–35) have been found. The authors have suggested that* E. senticosus* may be a candidate useful for developing therapeutic drugs for Alzheimer's disease. But authors have not provided any explanation for the possible mechanism of the extract's action and the influence on AChE and BuChE activity. The studies of the other research group showed that* E. senticosus* improves mental process in rats and reduces hippocampal CA1 neuronal death [13, 14]. Nevertheless, no further information on the inhibitory activity of remaining species (*E. setchuenensis*,* E. divaricatus*,* E. henryi*,* E. sessiliflorus*, and* E. gracilistylus*) towards AChE and BuChE has been found.

On the basis of Załuski's previous studies, it is concluded that these plants are a very promising source of biologically active substances. They inhibit metalloproteinases (MMPs, belonging to the hydrolase enzyme family, like AChE and BuChE) and neutralize free radicals. It was established that MMPs and free radicals take part in the etiopathogenesis of Alzheimer's disease as well. Some authors have reported on the damaging effects of ROS on neurones and on PD, AD, and ALS development. The oxidative stress is involved in the propagation of cellular injury that leads to neuropathology [15, 16].

The discovery of new inhibitors of AChE and BuChE is a highly attractive target both scientifically and commercially; for these reasons, there is a need to examine anti-AChE and anti-BuChE activity of* Eleutherococcus* species. To our knowledge, there are no such phytochemical reports concerning these cultivars. As part of a program to search for bioactive constituents from* Eleutherococcus* species, this study was focused on the establishment of anti-AChE and anti-BuChE activities of six* Eleutherococcus* species and aimed to discuss the possible antienzymatic mechanism and the structure-activity relationship. The work aimed at searching for antioxidants as well.

## 2. Materials and Methods

### 2.1. Standards and Reagents

Physostigmine, Ellman's reagent (DTNB, 5,5′-dithiobis(2-nitrobenzoic acid)), ATCh (acetylthiocholine iodide), BTCh (S-butyrylthiocholine iodide), AChE (acetylcholinesterase,* Electrophorus electricus*), BuChE (human plasma butyrylcholinesterase), sodium phosphate buffer, pH 7.0, and DPPH (2,2-diphenyl-1-picrylhydrazyl) were obtained from Sigma-Aldrich. Ethanol was obtained from POCH (Lublin, Poland). All other reagents were of analytical grade.

### 2.2. Plant Material

The roots of* E. senticosus* (Rupr. et Maxim.) Maxim.,* E. divaricatus* (Siebold et Zucc.) S. Y. Hu,* E. setchuenensis* (Harms) Nakai,* E. sessiliflorus* (Rupr. & Maxim.) S. Y. Hu,* E. gracilistylus* (W. W. Smith) S. Y. Hu, and* E. henryi* Oliv. were collected at the arboretum in Rogów (Poland) in October 2015 (voucher specimen numbers: ES01, ED02, ESetch03, ESes04, EG05, and EH06). All plant samples were deposited at the Department of Pharmacognosy, Collegium Medicum, Jagiellonian University, Cracow, Poland.

### 2.3. Dried Material Extraction with 75% Ethanol

The air-dried roots (5 g each) were soaked in 50 mL 75% ethanol for 24 h. Next, the samples were subjected to triple UAE type extraction (ultrasonic bath, Polsonic, Warsaw, Poland) using 1 × 50 mL and 2 × 25 mL of 75% ethanol. The extraction was performed at room temperature for 15 min for each cycle. Finally, 100 mL of each extract was obtained. The solvents were dried with an evaporator under vacuum conditions at 45°C and subjected to lyophilisation.

### 2.4. Dried Material Extraction with Chloroform

The roots were extracted in the same way as extraction with 75% ethanol using chloroform.

### 2.5. Antienzymatic Studies: Antiacetylcholinesterase and Antibutyrylcholinesterase Activities

The inhibition of AChE and BuChE was determined by the spectrophotometric method of Ellman et al. [17], and absorbance was measured on a multidetection BioTek spectrophotometer. The enzyme activity was 5 U/mg protein, and concentration of ATCh and BTCh was 15 mmol, DTNB (0.15 mmol). The extracts concentrations were 0.1, 0.5, 1.0, and 1.5 mg/mL in 10% water ethanol solution. The final concentrations in the reaction's mixture were 2.2, 11, 22, and 33 *μ*g/0.195 mL. Physostigmine was used as positive control at the following concentrations: 2, 3, 4, 15, 30, and 40 *μ*g/0.195 mL. Every assay was done in triplicate.

### 2.6. HPTLC Screening for Inhibitors of AChE and BuChE: Direct Bioautography (DB) Technique

HPTLC was performed using the method described by Załuski et al. [12]. Si_60_ HPTLC F_254_ plates and the following mobile phases were used: chloroform : methanol : water 70 : 30 : 4 v/v/v for the ethanol extracts and chloroform : diethyl ether 1 : 1 v/v for the chloroform extracts. The plates were developed for a distance of 90 mm. After drying at room temperature, the plates were dipped into the solution of AChE or BuChE (5 s) and next into Ellman's reagent, redried, and incubated for 30 min at 37°C in humid atmosphere. Subsequently, the chromatoplates were immersed for 5 s in the dipping solution of substrates (acetylthiocholine). After 3 min, white spots for AChE and BuChE inhibiting compounds should be detected on a yellow (in Ellman's reaction) background. The enzyme activity was 5 U/mg protein, and the concentration of ACh and BuCh was 15 mmol, DTNB (0.15 mmol).

### 2.7. HPTLC Screening for Antioxidants: Direct Bioautography (DB) Technique

TLC was done according to the method of Załuski et al. [12]. Briefly, TLC was performed on 10 cm × 20 cm glass Si60 HPTLC_F254_ plates using chloroform : methanol : water 70 : 30 : 4 v/v/v for the ethanol extracts and chloroform : diethyl ether 1 : 1 v/v for the chloroform extracts. The plate was developed for a distance of 90 mm. The plate was dried at room temperature for 20 min. After this time, the plate was immersed into 0.5% DPPH solution for 5 sec. Active compounds appeared as yellow-white spots against a purple background. White spots were visualized under day light after 1 min, 30 min, 1 h, 2 h, 5 h, 10 h, and 24 h.

### 2.8. Statistical Analysis

Determinations were performed in triplicate. The obtained data were subjected to statistical analysis using Statistica 7.0. (StatSoft, Cracow). The evaluations were analyzed for one-factor variance analysis. Statistical differences between the treatment groups were estimated by Spearman's (*R*) and Pearson's (*r*) test. All statistical tests were carried out at a significance level of *α* = 0.05.

## 3. Results and Discussion

### 3.1. Antienzymatic Activity

Various studies were performed concerning anticholinesterase activity of plant extracts or isolated compounds; however, up to now, most of the studies were focused on anti-AChE activity, not BuChE. Some of them are used to minimize the symptoms of neurodegenerative disorders, such as AD. Presently, several referenced natural acetylcholinesterase inhibitors are known, including physostigmine, galantamine, and huperzine A. The best known natural inhibitor of AChE is pyrroloindole alkaloid physostigmine isolated from* Physostigma venenosum*. Unfortunately, its use is limited due to poor intestinal absorption in humans. Another alkaloid, galantamine (Reminyl), isolated from* Galanthus nivalis* and* Leucojum vernum* is the additive to drugs used for the treatment of AD and was also licensed for use in early stages of AD in 2001. Their ingredients include plants extracts or pure isolates. A large number of alkaloids have been found to inhibit* in vitro* AChE. Studies on a variety of alkaloids have been performed focusing mainly on AChE activity, not BuChE. However, BuChE plays an important role as AChE, and the inhibition of just AChE can lead to a compensation mechanism and increased activity of the other one [6, 18]. Therefore, there is a need to search for inhibitors not only of AChE, but also of BuChE. Apart from enzymes activity, the second factor having an impact on neurodegenerative diseases is oxidative stress, which leads to neuronal damage.

In ancient times, people used to believe that* Eleutherococcus* species, especially* E. senticosus*, may have an impact on prevention of memory impairment or treatment of memory disorders. To give these observations a scientific basis, studies in this work aimed at the searching for both inhibitors AChE and BuChE and antioxidants in 12 extracts of six* Eleutherococcus* species (polar and nonpolar constituents).

The IC_50_ values for extracts measured by the microplate assay are shown in Table 1. In order to compare the antienzymatic activities of the extracts analyzed, physostigmine was used as the standard compound because of its well recognized activity. Physostigmine inhibited AChE in a dose-dependent way and 100% of inhibition was observed at concentration 30 *μ*g/195 *μ*L of the reaction mixture with IC_50_ value of 5 *μ*g.

The ethanol extracts of* E. setchuenensis* and* E. sessiliflorus* showed the strongest inhibition towards AChE (IC_50_: 0.3 and 0.3 mg/mL, resp.); among chloroform extracts, the most active appeared to be* E. gracilistylus* (IC_50_: 0.37 mg/mL). In turn, the ethanol extract of* E. henryi* was the strongest inhibitor towards BuChE with IC_50_ value of 0.13 mg/mL. Among chloroform extracts,* E. gracilistylus*,* E. setchuenensis*, and* E. sessiliflorus* appeared to be the strongest with IC_50_ values of 0.12, 0.18, and 0.19 mg/mL. According to Vinutha et al., plant extracts with an anti-AChE activity can be classified into 3 groups: potent group (>50% inhibition), moderate group (30–50% inhibition), and low activity group (<30% inhibition) [19].

Plant-based inhibitors of AChE and BuChE have been found in different plant families, such as Malvaceae, Rutaceae, Asteraceae, Fumariaceae, Papaveraceae, Lamiaceae, or Coniferaceae. According to scientific reports, some species used in traditional European medicine demonstrate inhibitory activity towards both AChE and BuChE. In screening studies, 12 species of plants have been used as remedies for the central nervous system. Of the 24 plant extracts (methanol and hexane), it was found that* Arnica chamissonis* Less. subsp.* foliosa* (Asteraceae) and* Ruta graveolens* L. (Rutaceae) had significant dose-dependent inhibition in the range between 29 and 88 *μ*g/mL (IC_50_ dose). But this effect was less than of the references galantamine and physostigmine. Other species have not had any significant AChE and BuChE inhibitory activity. Comparing the results obtained in this work to those cited above, it may be concluded that the chloroform extract from the roots of* E. setchuenensis*,* E. sessiliflorus*, and* E. gracilistylus* exhibited similar activity, towards BuChE, to the hexane extract from the herb of* Ruta graveolens* and the flowers of* Arnica chamissonis*. The 50% of inhibition was observed for the extract concentrations of 61 and 88 *μ*g/mL, respectively. In turn, the methanolic extracts from the herb of* Ruta graveolens* and the flowers of* Arnica chamissonis* showed stronger inhibition towards AChE than some* Eleutherococcus* species (IC_50_: 34 and 29 *μ*g/mL, resp.) [20]. The results of other studies have shown that from 180 medicinal plants only flavonoids isolated from* Agrimonia pilosa *Ledeb. inhibited AChE significantly [21]. Based on the results of already published studies, of 32 tested plants, the methanolic extracts from the roots of* Stephania suberosa* (L. L. Forman) and* Tabernaemontana divaricata* L. have shown high inhibitory activity, above 90%, at concentration of 0.1 mg/mL. In turn, the extracts from stems of* Piper interruptum* Opiz., seeds of* Piper nigrum* L., rootbarks of* Butea superba* Roxb., and roots of* Cassia fistula* L. showed 50–65% inhibitory activity on AChE at concentration of 0.1 mg/mL [22]. Biju et al. reported on anti-AChE activity of the ethanol extracts from* Baliospermum montanum*,* Humboldtia brunonis* Wall. var.* raktapushpa*, and* Pittosporum viridulum*. The IC_50_ values obtained in this study were a little bit lower than those obtained in the presented work (137.5, 105.7, and 128.3 *μ*g/mL for* Baliospermum montanum*,* Humboldtia brunonis*, and* Pittosporum viridulum*, resp.) [23]. The latest studies on the inhibition of AChE and BChE by the ethanol extracts from the leaves, stems, and flowers of* Euphorbia characias* subsp.* characias* revealed a weak effect with the IC_50_ values in the range between 0.6 and 5.8 mg/mL for AChE and 0.3 and 1.2 for BuChE, respectively. The IC_50_ values were, in some cases, ten orders of magnitude higher compared to the results obtained in this report [24]. According to Kaufmann et al., the methanolic extracts from* Berberis bealei* and* Phellodendron chinense* showed low IC_50_ values towards AChE (34.1 and 8.03 mg/mL, resp.). Despite the fact that these plants contain the alkaloids berberine, coptisine, and palmatine as the predominant compounds, their activity was weaker in comparison to the obtained results [25]. The AChE and BuChE inhibitory activity of the roots, leaves, and stem of* Jatropha gossypifolia* has been described. It appeared that the leaves ethyl acetate extract and the roots dichloromethane extract inhibited AChE the strongest with IC_50_ values 95.7 and 176.3 *μ*g/mL. The roots dichloromethane extract inhibited BuChE with IC_50_ value 77.6 *μ*g/mL [26]. Taking into consideration anti-AChE and anti-BuChE activities of plants extracts, some authors state that such an activity is dependent on all compounds which act synergistically. Therefore, plant extracts are very often used alone or as a combination with synthetic drugs to reduce side effects or maximize therapeutic outcomes.

**Figure 1:**
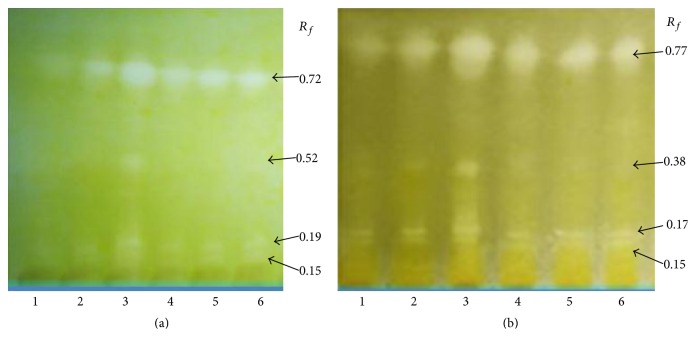
Screening of ethanol extracts of* Eleutherococcus* for AChE (a) and BuChE (b) inhibitors using the HPTLC assay method. White spots, corresponding to *R*_*f*_ values, on the yellow background represent the inhibition. (1)* E. senticosus*, (2)* E. divaricatus*, (3)* E. setchuenensis*, (4)* E. sessiliflorus*, (5)* E. henryi*, and (6)* E. gracilistylus*.

**Figure 2:**
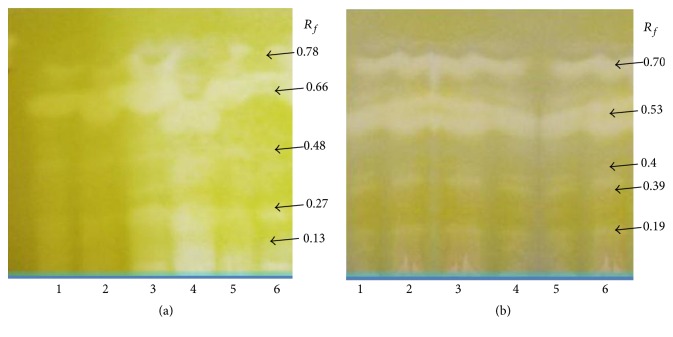
Screening of chloroform extracts of* Eleutherococcus* for AChE (a) and BuChE (b) inhibitors using the HPTLC assay method. White spots, corresponding to *R*_*f*_ values, on the yellow background represent the inhibition. (1)* E. senticosus*, (2)* E. divaricatus*, (3)* E. setchuenensis*, (4)* E. sessiliflorus*, (5)* E. henryi*, and (6)* E. gracilistylus*.

In the next step, the bioautography test was used to identify single potential inhibitors. The extracts were developed on HPTLC plates with the solvent system chloroform : methanol : water 70 : 30 : 4 v/v/v for the ethanol extracts and chloroform : diethyl ether 1 : 1 v/v for the chloroform extracts. The use of TLC screening for plant-based enzyme inhibitors is a rapid method and is free of disturbances due to the solvent. The AChE and BuChE inhibiting spots seen after spraying the substrate and enzyme are shown in Figures 1 and 2. All extracts have had AChE and BuChE inhibitors, which appeared as white spots on a pale yellow background. The same compounds have been identified as inhibitors of AChE and BuChE in the ethanol extracts at the *R*_*f*_ value as indicated in Figure 1. Each of the ethanol extracts had 4 inhibitors with *R*_*f*_ values 0.15, 0.19, 0.52, and 0.72 for AChE and 0.15, 0.17, 0.38, and 0.77 for BuChE. Considering chloroform extracts, 5 compounds in each extract have been identified to be potential inhibitors of AChE (*R*_*f*_ values 0.13, 0.27, 0.48, 0.66, and 0.78) and BuChE (*R*_*f*_ values 0.19, 0.39, 0.4, 0.53, and 0.70).

### 3.2. HPTLC-DB of Antioxidants

**Figure 3:**
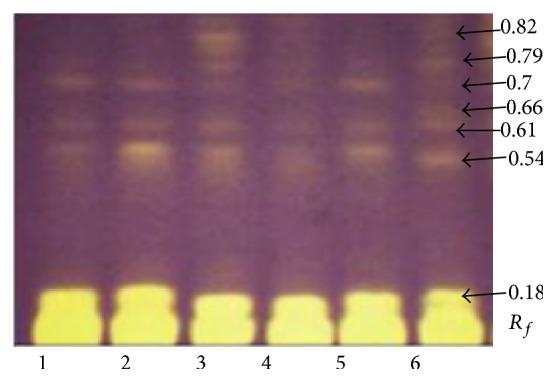
Screening of ethanol extracts of* Eleutherococcus* for anti-DPPH^*∗*^ scavengers using the HPTLC assay method. Yellow spots, corresponding to *R*_*f*_ values, on the purple background represent the inhibition. (1)* E. senticosus*, (2)* E. divaricatus*, (3)* E. setchuenensis*, (4)* E. sessiliflorus*, (5)* E. henryi*, and (6)* E. gracilistylus*.

**Figure 4:**
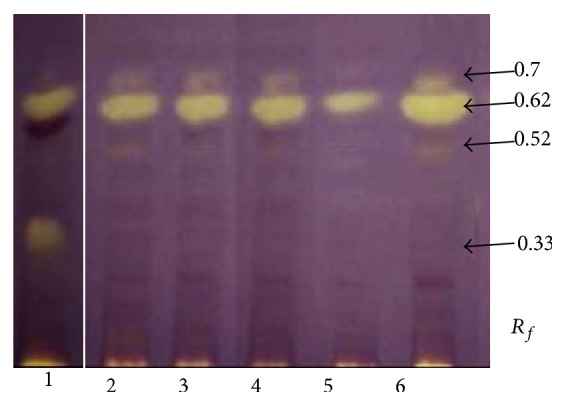
Screening of chloroform extracts of* Eleutherococcus* for anti-DPPH^*∗*^ scavengers using the HPTLC assay method. Yellow spots, corresponding to *R*_*f*_ values, on the purple background represent the inhibition. (1)* E. senticosus*, (2)* E. divaricatus*, (3)* E. setchuenensis*, (4)* E. sessiliflorus*, (5)* E. henryi*, and (6)* E. gracilistylus*.

Since many of the constituents of herbal extracts possess an antioxidative capacity, it is believed that this property may be involved, at least in part, in the antineurodegenerative mechanism of the herbal extract. The second part of the experiment was focused on the chromatographic identification of DPPH^*∗*^ scavengers, based partially on the fingerprinting conditions and results obtained by Załuski et al. [12]. The results are presented in Figures 3 and 4, as well as in Tables 2 and 3. Compounds that exhibit antiradical potential show up as yellow spots against a purple background. We observed the plates after 1 min, 30 min, 1 h, 2 h, 5 h, 10 h, and 24 h from the time of immersion of the plate in 0.5%  DPPH^*∗*^ solution. After 1 min, the ethanol extracts showed areas of activity on high *R*_*f*_ of 0.18 and 0.54. The compound at *R*_*f*_ 0.18 appeared as a strong-yellow spot, which correlates with its high anti-DPPH^*∗*^ activity. An additional yellow-coloured compound was observed to migrate at *R*_*f*_ of 0.61 and 0.7. Three other yellow spots at *R*_*f*_ values of 0.66, 0.79, and 0.82 appeared after 10 h. With all the investigated extracts, the richest in antioxidants are of* E. henryi* and* E. gracilistylus* (8 compounds). The results for all species remain in good agreement with the data referring to the spectrophotometric DPPH^*∗*^ assay method. Załuski et al. exhibited good radical scavenging activity of the ethanol extracts with EC_50_ values between 1.2 and 3.5 mg/mL [11].

Taking into account the speed of DPPH^*∗*^ decolourization by chloroform extracts, the differences have been noticed. Because of the presence of nonpolar compounds, the chloroform extracts exhibited a smaller number of inhibition zones. After 1 min, the extracts showed areas of activity on high *R*_*f*_ of 0.33, 0.52, 0.62, and 0.7. In all the analyzed species, compounds at *R*_*f*_ values of 0.52, 0.62, and 0.7 were detected. Among them, the compound at *R*_*f*_ value of 0.62 appeared as the most intensive yellow band.

### 3.3. Chemical Backgrounds of Anti-AChE, Anti-BuChE, and Antioxidant Activity of the Analyzed Extracts

This work has been a continuation of the work on phytochemistry and bioactivity of* Eleutherococcus* species since 2008. The chemical profile of the species includes mainly polyphenols, of which the most abundant are eleutherosides B, E, and E1. This group of compounds is thought to be the most pharmacologically active. In Załuski's previous studies on* Eleutherococcus*, it was revealed that the analyzed species contain eleutherosides B, E, and E1, even in a larger amount than what the Polish or European Pharmacopoeia requires. According to the Polish Pharmacopoeia monograph, the root of* E. senticosus* should contain minimum 0.08% for the sum of eleutheroside B and eleutheroside E. On the basis of the HPTLC-densitometric assay, the amount of eleutheroside B ranged from 0.08 to 0.34%; that of eleutheroside E from 0.05 to 0.13%; and that of eleutheroside E1 from 0.02 to 0.09%. Another compound is (+)-sesamin, which was isolated and identified by means of GC-MS, ^1^H- and ^13^C-NMR, and two-dimensional NMR experiments (HMQC, HMBC, COSY, and DEPT) [10, 27].

The second group of compounds present in the roots of* Eleutherococcus* species are essential oil and aroma components. (*E,E*)-Farnesol, an acyclic sesquiterpene alcohol, was the major constituent up to 43.6%, followed by *α*-pinene (18.8%) and pentadecanoic acids (12.3%), [28].

All above compounds contain in their structure the chemical groups that can interact with the active site of AChE and BuChE and can scavenge free radicals. They can act as chelators of metal ions playing as cofactors for an enzyme activity. The next possible mechanism is the interactions between active groups of inhibitors and amino acids of the active site of enzymes. However, further detailed studies on the mechanism of inhibition will provide reliable results.

In case of anti-DPPH^*∗*^ activity, Załuski at el.'s previous studies revealed that, in this activity, eleutheroside E1 can be involved. This results from its molecular structure that demonstrates the one free OH group in the phenyl ring and four methoxy groups (two in each aromatic ring). This was called “Załuski's eleutheroside hypothesis” [9].

## 4. **Conclusion**

These basic studies on compounds present in* Eleutherococcus* species contribute to the development of general knowledge about plant-based drugs. The study confirms confirms the rightness of the traditional use of these species in prevention or treatment of memory impairment and gives useful information for further purifying steps to dereplicate active compounds. Further, more detailed studies will be done on spectral identification of isolates and in* in vivo* models.

## Figures and Tables

**Table 1:** IC_50_ values for extracts (mg/mL). NA: not active.

Species	75% ethanol		Chloroform	
	AChE	BuChE	AChE	BuChE
*E. senticosus*	0.46 ± 0.04	0.73 ± 0.1	0.75 ± 0.08	0.71 ± 0.08
*E. divaricatus*	0.94 ± 0.09	0.54 ± 0.04	NA	1.68 ± 0.3
*E. setchuenensis*	0.3 ± 0.06	0.95 ± 0.2	1.08 ± 0.1	0.18 ± 0.01
*E. sessiliflorus*	0.3 ± 0.06	0.59 ± 0.04	NA	0.19 ± 0.01
*E. henryi*	1.75 ± 0.1	0.13 ± 0.02	NA	1.21 ± 0.2
*E. gracilistylus*	0.41 ± 0.02	1.22 ± 0.3	0.37 ± 0.05	0.12 ± 0.04

**Table 2:** Comparison of *R*_*f*_ values and a number of compounds for free radical scavenging HPTLC fingerprints for the ethanol extracts.

Sample	*R*_*f*_								Number of compounds
	0.18	0.54	0.61	0.66	0.70	0.76	0.79	0.82	
*E. senticosus*	+	+	+		+	+			5
*E. divaricatus*	+	+	+		+	+			5
*E. setchuenensis*	+	+	+		+	+	+	+	7
*E. sessiliflorus*	+	+	+		+	+			5
*E. henryi*	+	+	+	+	+	+	+	+	8
*E. gracilistylus*	+	+	+	+	+	+	+	+	8

**Table 3:** Comparison of *R*_*f*_ values and a number of compounds for free radical scavenging HPTLC fingerprints for the chloroform extracts.

Sample	*R*_*f*_				Number of compounds
	0.33	0.52	0.62	0.70	
*E. senticosus*	+	+	+	+	4
*E. divaricatus*	+	+	+	+	4
*E. setchuenensis*		+	+	+	3
*E. sessiliflorus*		+	+	+	3
*E. henryi*		+	+	+	3
*E. gracilistylus*		+	+	+	3
